# Evaluation of the relationship between dietary acid load and cardiovascular risk factors in patients with type 2 diabetes: a case–control study

**DOI:** 10.3389/fnut.2024.1445933

**Published:** 2024-08-14

**Authors:** Sedef Güngör, Mendane Saka

**Affiliations:** ^1^Faculty of Health Science, Department of Nutrition and Dietetics, Atılım University, Ankara, Türkiye; ^2^Faculty of Health Science, Department of Nutrition and Dietetics, Başkent University, Ankara, Türkiye

**Keywords:** cardiovascular disease, dietary acid load, type 2 diabetes, potential renal acid load, net endogenous acid production

## Abstract

**Backround:**

Diets high in dietary acid load are thought to be associated with metabolic diseases. However, the number of studies examining the relationship between dietary acid load and metabolic diseases in Turkey is insufficient. The aim of this study was to investigate the relationship between cardiovascular disease risk factors and dietary acid load in individuals with type 2 diabetes.

**Materials and methods:**

In this case–control study, 51 participants aged 30–65 years with type 2 diabetes and 59 participants in the control group were included. Blood pressure and biochemical findings were measured. Anthropometric measurements and body composition measurements were made. Dietary intake was assessed using a 3-day (1 day on weekends, 2 days on weekdays) food consumption record. Dietary acid load scores, including potential renal acid load (PRAL) and net endogenous acid production (NEAP), were calculated based on dietary intake. NEAP and PRAL scores were categorized as low and high according to the median value. Smoking status, body mass index (BMI), systolic blood pressure (SBP), diastolic blood pressure (DBP), total cholesterol (TC), trigylceride (TG), high density lipoprotein cholesterol (HDL-C), low density lipoprotein cholesterol (LDL), waist-hip ratio (WHR), waist-to- height ratio (WtHR), hemoglobin and fat mass (%) were evaluated as cardiovascular risk factors.

**Results:**

The cut-off values of PRAL and NEAP were 3.61 and 44.78 mEq/d, respectively. After adjustment for various covariates, a significant positive association between PRAL and TG levels was observed in the diabetic group [odds ratio (OR), 5.98; 95% CI, 1.45–24.67; *p* = 0.013]. In contrast, a negative association was found between PRAL and SBP in the control group [odds ratio (OR), 0.21; 95% CI, 0.05–0.83; *p* = 0.026]. However, these associations were not observed for NEAP values in either group.

**Conslusions:**

A higher PRAL value was consistently associated with higher TG level, but other cardiovascular risk factors were not. More longitudinal and interventional studies are needed to better establish a causal effect between dietary acid load and cardiovascular risk factors in individuals with diabetes.

## Introduction

Diabetes is considered one of the most significant health problems of today. According to the Diabetes Atlas, there were 537 million adult diabetes patients worldwide in 2021, and it is estimated that this number will reach 783 million by 2045. In addition, Turkey has the highest diabetes population among European countries (1). Diabetes is a global health problem with a rapidly increasing prevalence. It is a chronic disease characterized by high blood glucose levels and abnormalities of carbohydrate, fat and protein metabolism (2). Chronic hyperglycemia caused by diabetes can cause microvascular and macrovascular complications in the long term.

Diabetes is an important risk factor for cardiovascular disease (CVD) (3). CVD risk factors such as obesity, hypertension, and dyslipidemia are common in patients with diabetes, especially those with type 2 diabetes (4). It is important to determine the risk of CVD, including diabetes, in the adult age group because CVD and diabetes generally affect each other, many risk factors are common, and more than one risk factor occurs together in individuals during adulthood (5). The management of modifiable CVD risk factors such as hyperglycemia, dyslipidemia, obesity, unhealthy diet, and physical inactivity is critically important to minimize the risk of macrovascular complications of diabetes (3).

Altering dietary habits is an important strategy in managing and preventing CVD risk factors. Food intake can affect’s the body’s acid base balance through the intake of acid or base precursors. Sulfur amino acids, which are the main determinants of acid load in the diet, are found in high amounts in foods of animal origin such as meat, eggs, fish and cheese, on the other hand, potassium and magnesium in plant foods and calcium in both plant foods and dairy products are determinants of alkaline load. A diet high in animal products and other acid-producing foods can lead to an acid load that cannot be compensated by fruit and vegetable consumption. This can lead to diet-induced metabolic acidosis (6). Recent studies have focused on the association between dietary acid load and health-related outcomes, including cardiometabolic risk factors and diabetes (6–11). It is thought that even a small reduction in diet-induced metabolic acidosis may improve insulin sensitivity, thus reducing the acid load in the diet may be effective in reducing insulin resistance (7). In this study, it was determined that the dietary acid loads of 125 newly diagnosed diabetic individuals were similar compared to the control group. There are studies showing that increased dietary acid load may be positively associated with insulin resistance that may develop in the future and may increase the risk of diabetes (6, 11–13). While a Korean study put forward that dietary acid load was positively associated with the development of insulin resistance in the future (12), a longitudinal study by Moghadam et al. (9) in Iran emphasized that high dietary acid–base load may be a risk factor for the development of insulin resistance and related metabolic disorders.

The acid-forming potential of foods can be calculated using potential renal acid load (PRAL) and net endogenous acid production (NEAP). PRAL, developed by Remer et al. (14), takes into account different intestinal absorption rates of nutrients, ionic balances for calcium and magnesium, and dissociation of phosphate at pH 7.4. A positive PRAL score reflects acid-forming potential, while a negative score indicates alkaline-forming potential. Frassetto and colleagues (15) proposed a computational model focusing on (total) protein and potassium, which are thought to be the main variables responsible for NEAP. These methods are used to estimate acid loads from food intake and are frequently used in epidemiological studies. Because dietary acid load is related to urinary acid load measured from 24-h urine, it provides a simple and useful tool to assess the acidity of the diet (14, 15).

The number of studies examining the relationship between diabetes, CVD and dietary acid load is limited. In addition, studies examining the relationship between diabetes, cardiometabolic risk factors and dietary acid load are inconsistent. Therefore, the aim of this study is to examine the relationship between dietary acid load and cardiometabolic risk factors in patient with diabetes.

## Materials and methods

### Study design and participant

In this case–control study, participants aged 30–65 years with a diagnosis of Type 2 diabetes according to the American Diabetes Association criteria and age- and gender-matched controls who applied to Ankara Başkent University Hospital Endocrinology and Metabolic Diseases Outpatient Clinic between November 2019 and December 2020 were included. Diabetic patients with a history of any chronic disease such as CVD, cancer (including those with a history), kidney disease, gastrointestinal disorders and liver and lung diseases, acute infection, following any special diet or physical activity, daily energy intake outside the 800–4,200 kcal range, as well as pregnant and lactating patients were excluded. These patients who applied to the outpatient clinic and met our criteria were included in our study. The control group was selected from patients residing in Ankara, who had blood glucose control within the last 6 months and met the exclusion criteria. Exclusion criteria for the control group are as follows: participants with a history of any chronic disease such as CVD, cancer (including those with a history), kidney disease, gastrointestinal disorders and liver and lung diseases, acute infection, adherence to a specific lifestyle (diet and/or physical activity), medication use that may affect weight and diet, pregnant and lactating mothers, and daily energy intake outside the 800–4,200 kcal range were excluded. Urine albumin-to-creatinine ratio and estimated glomerular filtration rate (eGFR) were analyzed to assess renal function in individuals thought to be affected by dietary acid load. Participants with urine albumin-creatinine ratio > 30 mg/g and eGFR <60 mL/min/1.73 m2 were also excluded from the study. eGFR was calculated using the chronic kidney disease epidemiology collaboration equation (CKD-EPI equation, http://www.nkdep.nih.gov). Sixty people in the diabetes group and 64 people in the control group were included in the study. Participants with high urine-albumin creatinine levels, low eGFR levels, and participants whose body composition measurement data and food consumption records could not be obtained due to the pandemic were excluded from this study (A total of 9 people in the diabetes group and 5 people in the control group were not included in the study). Accordingly, the study was conducted with 51 people in the diabetes group and 59 people in the control group.

### Biochemical parameters

All laboratory assessments were measured after a 10–12 h overnight fast. The blood pressures and biochemical findings of the patients were taken by the nurse working in the hospital. Fasting blood glucose (FBG), low-density lipoprotein (LDL-C) and high-density lipoprotein (HDL-C) cholesterol, total cholesterol (TC), triglyceride (TG), hemoglobin, serum creatinine, eGFR and urine albumin/creatinine values were collected from biochemical test values routinely obtained at Başkent University Ankara Hospital. The fasting blood glucose collected was used to confirm that individuals in the control group did not have prediabetes or diabetes. The biochemical findings of the individuals who accepted the study were obtained from the medical records. Blood pressure (mmHg) was measured from the left arm using a mercury manometer while the person was sitting and calm.

Hypertension [systolic blood pressure (SBP) ≥ 130 mm Hg and diastolic blood pressure (DBP) ≥ 85 mm Hg], blood lipids [dyslipidemia LDL-C (≥130 mg/dL), HDL-C (male <40 mg/dL, female <50 mg/dL), TG (≥150 mg/dL)], were evaluated according to the National Cholesterol Education Program, Adult Treatment Panel III diagnostic criteria (16).

### Assessment of other variables

Demographic information (age, sex, marital status, smoking and education level, etc.) was collected by face-to-face interviews with the participants, anthropometric measurements were made and a 3-day food consumption record (1 day on weekends, 2 days on weekdays) was obtained.

Body weight was measured while wearing light clothing and without shoes using the TANITA TBF-300 (TANITA Corp., Tokyo, Japan) body composition monitoring scale. Body fat mass (FM) percentage and body fat free mass (FFM) percentage were obtained using TANITA. Body height was measured using a tape meter (Seca scale; Seca Hamburg, Germany) in a standing position without shoes, while the shoulders were in normal position. Body Mass Index (BMI) (kg/m^2^) was calculated by dividing weight in kilograms by the square of height in meters. BMI was defined according to cut-off values reported by the World Health Organization (WHO; overweight and obesity: BMI ≥25 kg/m^2^) (17). Waist circumference (WC) and hip circumstance (HC) were measured with an accuracy of 0.1 cm using standard methods by tape measure without any pressure to the body surface. The waist-hip ratio (WHR) was also calculated by dividing WC by HC. Waist-to-height ratio (WtHR) was also calculated by dividing WC by height. All measurements were obtained as described previously and taken by a trained dietician.

### Dietary assessment and definition of dietary acid load

In order to evaluate the daily energy and nutrients in the diet and to calculate the dietary acid load, 3-day 24-h food consumption records were taken from the individuals participating in the study, 2 days on weekdays and 1 on weekends. The daily diet, energy and nutrient intake from these data were analyzed using the “Computer Assisted Nutrition Program, Nutrition Information Systems Package Program (BEBIS)” developed for Turkey.

Various formulas have been used recently to estimate dietary acid load. The first is a physiological-based computational model used to estimate PRAL of foods. This model predicts endogenous acid production exceeded alkaline production for a certain amount of nutrients ingested daily (14, 15).

PRAL was calculated using the following algorithm:


$$\begin{array}{l} \;\mathrm{PRAL}\;\left(\mathrm{mEq}/d\right)=0.49\;x\;\mathrm{protein}\;\mathrm{intake}\;\left(g/\mathrm{day}\right)+\\ 0.037\;x\;\mathrm{phosphorus}\;\left(\mathrm{mg}/\mathrm{day}\right)-0.021\;x\;\mathrm{potassium}\;\left(\mathrm{mg}/\mathrm{day}\right)\\ -0.013\;x\;\mathrm{calcium}\;\left(\mathrm{mg}/\mathrm{day}\right)-0.026\;x\;\mathrm{magnesium}\;\left(\mathrm{mg}/\mathrm{day}\right)\text{.} \end{array}$$

The calculation formula of the NEAP value, which is the second model used to calculate the dietary acid load of foods, is shown below:


$$\begin{array}{l} \;\mathrm{NEAP}\;\left(\mathrm{mEq}/\mathrm{day}\right)\\ =\left[54.5\;x\;\mathrm{protein}\;\left(g/\mathrm{day}\right)/\mathrm{potassium}\;\left(\mathrm{mEq}/\mathrm{day}\right)\right]-10.2\text{.} \end{array}$$

### Statistical analysis

In the statistical analysis phase of the study, firstly, the results of the Shapiro–Wilk test were examined to test the conformity of the numerical variables to the assumption of normal distribution. “Independent samples t-test” was used for two-group comparisons that conformed to normal distribution, and ‘Mann–Whitney-U test’ was used for those that did not conform to normal distribution. “Pearson chi-square test” was used for grouped data. The relationships between group variables were examined by correlation analysis. While the correlation analysis was being applied, the expected observation values of the cells were taken into account. “Fisher test” was used in comparisons of the number of observations with expected observation values below 5, and “Pearson chi-square test” was used in cases where the expected observation value was greater than 5. Then, logistic regression analysis was applied with the variables found to be statistically significant. Shapiro–Wilk test results were examined to test the compliance of numerical variables with the normal distribution assumption. “Independent samples t-test” was used for two-group comparisons suitable for normal distribution. In the study, logistic regression analysis was applied to determine the factors affecting the groups of PRAL and NEAP variables. Groups whose PRAL and NEAP values were below the median (Q2) were classified as low level, and those above were classified as high level. Binary logistic regression analysis was applied, taking low-high level PRAL and NEAP groups as dependent variables. Logistic regression analysis Two different models were created as Model −1 (unadjusted model) and Model-2 (adjusted model). In the adjusted model, age, sex, marital status and BMI variables were controlled. A *p*-value of <0.05 was set as statistically significant. Findings regarding the hypothesis tests were obtained using the IBM SPSS 26 program. NOTE: During the regression analysis phase, it was determined that the FFM (%) variable caused a multicollinearity problem (OR > 24,000) and was disabled from the entire analysis.

## Results

The findings of the cases and controls included in the study are presented in Table 1. Considering these results, the average age of individuals in the diabetes group was 51.5 years, while the average age of individuals in the control group was 48.6 years (*p* > 0.05). Body fat (%), TG, SBP and DBP values of individuals in the diabetes group were significantly higher than those in the control group (*p* < 0.05). In addition, there was no significant difference between the groups in mean PRAL and NEAP values (p > 0.05) (Table 1). The average FM% of the individuals in the study is 34.9 ± 13.89 and the average hemoglobin values are 8.2 ± 1.37. The median value of PRAL is determined as 3.612, while the median value of NEAP is 44.783.

**Table 1:** Findings of individuals included in the research.

Variable	Diabetes group (*n* = 51)	Control group (*n* = 59)	*P*
**Sex, *n* (%)**			
Male	14 (27.5)	14 (23.7)	0.655*
Female	37 (72.5)	45 (76.3)	
**Age (years)**	51.5 ± 7.3	48.6 ± 8.5	0.086**
**Marital status, *n* (%)**			
Married	42 (82.4)	48 (81.4)	0.892*
Single	9 (17.6)	11 (18.6)	
**Smoking status, *n* (%)**			
Yes	18 (35.3)	20 (33.9)	0.922*
No	29 (56.9)	33 (55.9)	
Quit	4 (7.8)	6 (10.9)	
**BMI (kg/m**^**2**^**)**	31.4 ± 4.9	27.7 ± 3.8	0.067^‡^
**WHR**	0.95 ± 0.07	0.88 ± 0.08	0.154^‡^
**WtHR**	0.61 ± 0.07	0.55 ± 0.05	0.201^‡^
**FM (%)**	36.5 ± 9.4	33.4 ± 6.7	0.016**
**Hemoglobin (g/dL)**	13.9 ± 1.4	13.8 ± 1.3	0.384^‡^
**TG (mg/dL)**	164.6 ± 58.04	114.4 ± 51.9	<0.001**
**TC (mg/dL)**	229.9 ± 36.8	213.8 ± 47.2	0.147^‡^
**HDL-C (mg/dL)**	45.4 ± 11.2	50.4 ± 9.8	0.678^‡^
**LDL-C (mg/dL)**	151.9 ± 32.5	142.1 ± 40.9	0.238^‡^
**SBP (mmHg)**	128.5 ± 16.4	120.9 ± 10.3	0.021**
**DBP (mmHg)**	80.9 ± 7.7	76.03 ± 12.04	0.017**
**Physical activity, *n* (%)**			
Yes	14 (27.5)	24 (40.7)	0.146*
No	36 (72.5)	35 (59.3)	
**PRAL (mEq/day)**	2.1 ± 8.9	4.4 ± 9.4	0.899^‡^
**NEAP (mEq/day)**	45.8 ± 13.1	48.9 ± 13.7	0.363^‡^

Findings for categorical variables are shown as *n* (%).Numerical data are given as Mean ± Standard deviation.

BMI, body mass index; DBP, diastolic blood pressure; F, Fisher test; FM, fat mass; HDL-C, high density lipoprotein cholesterol; K, Pearson chi-square independence test; LDL-C low-density lipoprotein cholesterol; NEAP, net endogenous acid production; PRAL, potential renal acid load; SBP, systolic blood pressure; TC, total cholesterol; TG, triacylglycerol; WtHR, Waist-to-height ratio; WHR, waist-hip ratio.

* *p*-value is for Chi square test.

** *p*-value is for Mann–Whitney test.

‡ Independent *t* test.

The characteristics of the individuals in the diabetes and control groups and comparisons between PRAL groups are given in Table 2. There is a statistically significant relationship between sex, TG, HDL-C and PRAL groups of individuals with diabetes (*p* < 0.05). When these relationships are examined, it is seen that women tend to follow a diet with a low PRAL value. In the diabetes group, most of the individuals with TG values below 150 were found to have low PRAL values. In addition, the majority of diabetes individuals with high HDL-C values had low PRAL values. When the results of the control group were analyzed, there was a statistically significant relationship between PRAL value and only SBP and HDL-C (*p* < 0.05). No significant relationship was found between other cardiovascular risk factors. Most of the individuals in the control group with low SBP values had higher PRAL values. Furthermore, individuals with low HDL-C values in the control group tended to have PRAL values greater than 3.612.

**Table 2:** Relationships between PRAL and characteristics of individuals in the diabetes and control groups.

Variable	PRAL (mEq/day)					
	Diabetes group (*n* = 51)		p1	Control group (*n* = 59)		p2
	Low (<3.612)	High (>3.612)		Low (<3.612)	High (>3.612)	
**Sex, *n* (%)**						
Female	26 (%70.3)	11 (%29.7)	0.007 *^K^*	22 (%48.9)	23 (%51.1)	0.069 *^K^*
Male	4 (%28.6)	10 (%71.4)		3 (%21.4)	11 (%78.6)	
**Smoking status, *n* (%)**						
Yes	10 (%55.6)	8 (%44.4)	0.846*^F^*	8 (%40.0)	12 (%60.0)	0.829 *^K^*
No	18 (%62.1)	11 (%37.9)		15 (%45.5)	18 (%54.5)	
Quit	2 (%50.0)	2 (%50.0)		2 (%33.3)	4 (%66.7)	
**BMI (kg/m**^**2**^**), *n* (%)**						
<25	3 (%75.0)	1 (%25.0)	0.451*^F^*	5 (%31.3)	11 (%68.8)	0.225 *^K^*
≥25	27 (%57.4)	20 (%42.6)		20 (%46.5)	23 (%53.5)	
**SBP (mmHg), *n* (%)**						
<130	14 (%53.8)	12 (%46.2)	0.620 *^K^*	15 (%34.1)	29 (%65.9)	0.046*^F^*
≥130	14 (%60.9)	9 (%39.1)		9 (%64.3)	5 (%35.7)	
**DBP (mmHg), *n* (%)**						
<85	21 (%55.3)	17 (%44.7)	0.297*^F^*	21 (%39.6)	32 (%60.4)	0.285*^F^*
≥85	7 (%63.6)	4 (%36.4)		3 (%60.0)	2 (%40.0)	
**TC (mg/dL), *n* (%)**						
<200	7 (%63.6)	4 (%36.4)	0.222 *^K^*	11 (%51.4)	10 (%47.6)	0.221 *^K^*
≥200	23 (%60.5)	15 (%39.5)		13 (%35.1)	24 (%64.9)	
**TG (mg/dL), *n* (%)**						
<150	20 (%83.3)	4 (%16.7)	0.003 *^K^*	19 (%45.2)	23 (%54.8)	0.344*^F^*
≥150	10 (%38.5)	16 (%61.5)		6 (%35.3)	11 (%64.7)	
**HDL-C (mg/dL), *n* (%)**						
M < 40, *F* < 50	17 (%58.8)	12 (%41.2)	0.029 *^K^*	8 (%34.8)	15 (%65.2)	0.044 *^K^*
M ≥ 40, F ≥ 50	12 (%63.2)	7 (%36.8)		15 (%47.6)	19 (%52.4)	
**LDL-C (mg/dL), *n* (%)**						
<130	8 (%66.7)	4 (%33.3)	0.205 *^K^*	10 (%45.5)	12 (%54.5)	0.460*^F^*
≥130	22 (%59.5)	15 (%40.5)		15 (%40.5)	22 (%59.5)	
**WHR, *n* (%)**						
M < 0.90, *F* < 0.85	2 (%66.7)	1 (%33.3)	0.638*^F^*	12 (%46.2)	14 (%53.8)	0.398*^F^*
M ≥ 0.90, F ≥ 0.85	28 (%58.3)	20 (%41.7)		13 (%39.4)	20 (%60.6)	
**WtHR, *n* (%)**						
<0.50	1 (%100.0)	0 (%0.0)	0.588*^F^*	2 (%28.6)	5 (%71.4)	0.359*^F^*
≥0.50	29 (%58.8)	21 (%42.0)		23 (%44.2)	29 (%55.8)	
**Physical activity, *n* (%)**						
Yes	6 (%42.9)	8 (%57.1)	0.135*^F^*	7 (%35.0)	13 (%65.0)	0.295*^F^*
No	24 (%64.9)	13 (%35.1)		18 (%46.2)	21 (%53.8)	
**FM (%)**	38.93 ± 8.47	33.14 ± 9.86	0.029	33.94 ± 6.89	32.97 ± 6.58	0.586
**Hemoglobin (g/dL)**	13.71 ± 1.47	14.40 ± 1.35	0.095	13.72 ± 1.04	13.88 ± 1.49	0.646

Findings for categorical variables are shown as *n* (%).Numerical data are given as Mean ± Standard deviation.

BMI, body mass index; DBP, diastolic blood pressure; F, Fisher test; FM, fat mass; HDL-C, high density lipoprotein cholesterol; K, Pearson chi-square independence test; LDL-C low-density lipoprotein cholesterol; NEAP, net endogenous acid production; PRAL, potential renal acid load; SBP, systolic blood pressure; TC, total cholesterol; TG, triacylglycerol; WtHR, Waist-to-height ratio; WHR, waist-hip ratio.

The characteristics of individuals in the diabetes and control groups and comparisons between the NEAP groups were presented in Table 3. In both groups, there was no statistically significant relationship between NEAP value and smoking, BMI, SBP, DBP, TC, TG, LDL-C, WHR, WtHR, physical activity and haemoglobin values (*p* > 0.05). However, there is a statistically significant relationship between NEAP value and sex in both groups (*p* < 0.05). Men tended to eat diets with a high dietary acid load, while women tended to eat diets with a lower dietary acid load. In addition, when the results of the control group were analyzed, it was observed that the majority of individuals with low NEAP values had high HDL-C values (*p* < 0.05).

**Table 3:** Relationships between NEAP and characteristics of individuals in the diabetes and control groups.

Variable	NEAP (mEq/day)					
	Diabetes group (*n* = 51)		p1	Control group (*n* = 59)		p2
	Low (<44.783)	High (>44.783)		Low (<44.783)	High (>44.783)	
**Sex, *n* (%)**						
Female	25 (%67.6)	12 (%32.4)	0.014*^F^*	23 (%51.1)	22 (%48.9)	0.048*^F^*
Male	4 (%28.6)	10 (%71.4)		3 (%21.4)	11 (%78.6)	
**Smoking status, *n* (%)**						
Yes	8 (%44.4)	10 (%55.6)	0.351*^K^*	10 (%50.0)	10 (%50.0)	0.740 *^K^*
No	19 (%65.5)	10 (%34.5)		14 (%42.4)	19 (%57.6)	
Quit	2 (%50.0)	2 (%50.0)		2 (%33.3)	4 (%66.7)	
**BMI (kg/m**^**2**^**), *n* (%)**						
<25	3 (%75.0)	1 (%25.0)	0.417*^F^*	4 (%25.0)	12 (%75.0)	0.065*^F^*
≥25	26 (%55.3)	21 (%44.7)		22 (%51.2)	21 (%48.8)	
**SBP (mmHg), *n* (%)**						
<130	13 (%50.0)	13 (%50.0)	0.338 *^K^*	17 (%38.6)	27 (%61.4)	0.251 *^K^*
≥130	14 (%60.9)	9 (%39.1)		8 (%57.1)	6 (%42.9)	
**DBP (mmHg), *n* (%)**						
<85	21 (%55.3)	17 (%44.7)	0.454 *^K^*	22 (%41.5)	31 (%58.5)	0.382 *^K^*
≥85	6 (%54.5)	5 (%45.5)		3 (%60.0)	2 (%40.0)	
**TC (mg/dL), *n* (%)**						
<200	7 (%63.6)	4 (%36.4)	0.239 *^K^*	10 (%47.6)	11 (%52.4)	0.458 *^K^*
≥200	22 (%57.9)	16 (%42.1)		15 (%40.5)	22 (%59.5)	
**TG (mg/dL), *n* (%)**						
<150	17 (%70.8)	7 (%29.2)	0.108 *^K^*	21 (%50.0)	21 (%50.0)	0.124*^F^*
≥150	12 (%46.2)	14 (%53.8)		5 (%29.4)	12 (%70.6)	
**HDL-C (mg/dL), *n* (%)**						
M < 40, F < 50	19 (%63.3)	11 (%36.7)	0.128 *^K^*	7 (%30.4)	16 (%69.6)	0.036 *^K^*
M ≥ 40, F ≥ 50	10 (%52.6)	9 (%47.4)		17 (%50.0)	17 (%50.0)	
**LDL-C (mg/dL), *n* (%)**						
<130	7 (%58.3)	5 (%41.7)	0.253 *^K^*	9 (%40.9)	13 (%59.1)	0.459*^F^*
≥130	22 (%59.5)	15 (%40.5)		17 (%45.9)	20 (%54.1)	
**WHR, *n* (%)**						
M < 0.90, F < 0.85	2 (%66.7)	1 (%33.3)	0.604*^F^*	11 (%42.3)	15 (%57.7)	0.509*^F^*
M ≥ 0.90, F ≥ 0.85	27 (%56.3)	21 (%43.8)		15 (%45.5)	18 (%54.4)	
**WtHR, *n* (%)**						
<0.50	1 (%100.0)	0 (%0.0)	0.569*^F^*	1 (%14.3)	6 (%85.7)	0.097 *^K^*
≥0.50	28 (%56)	22 (%44)		25 (%48.1)	27 (%51.9)	
**Physical activity, *n* (%)**						
Yes	6 (%42.9)	8 (%57.1)	0.177*^F^*	9 (%45.0)	11 (%55.0)	0.567*^F^*
No	23 (%62.2)	14 (%37.8)		17 (%43.6)	22 (%56.4)	
**FM (%)**	38.82 ± 8.81	33.55 ± 9.56	0.046	34.83 ± 6.89	32.24 ± 6.36	0.139
**Hemoglobin (g/dL)**	13.67 ± 1.53	14.42 ± 1.25	0.068	13.64 ± 1.08	13.94 ± 1.47	0.392

Findings for categorical variables are shown as n (%).Numerical data are given as Mean ± Standard deviation.

BMI, body mass index; DBP, diastolic blood pressure; F, Fisher test; FM, fat mass; HDL-C, high density lipoprotein cholesterol; K, Pearson chi-square independence test; LDL-C low-density lipoprotein cholesterol; NEAP, net endogenous acid production; PRAL, potential renal acid load; SBP, systolic blood pressure; TC, total cholesterol; TG, triacylglycerol; WtHR, Waist-to-height ratio; WHR, waist-hip ratio.

The results of the regression model in which the PRAL variable was taken as the dependent variable were given in Table 4. When the findings of the diabetes group are examined, TG variable has a significant effect on the PRAL variable in both the unadjusted and adjusted models (*p* < 0.05). Individuals with TG higher than 150 are more likely to have high PRAL than individuals with low TG (OR = 5.983). This rate is approximately 2 times higher in the adjusted model (OR = 10.226). When the results of the diabetes group are examined, it is seen that HDL-C and FM (%) variables do not have a significant effect on the PRAL variable in the unadjusted and adjusted models. When the findings of the control group are examined, it is seen that SBP has a significant effect on the PRAL variable in both the unadjusted and adjusted models (*p* < 0.05). In the unadjusted model, individuals with SBP variable higher than 130 are 78.8% less likely to have high PRAL. In the adjusted model, this ratio increases even more. (83.2%). When the results of the control group are examined, it is seen that the HDL-C variable does not have a significant effect on PRAL in both models.

**Table 4:** Regression model with PRAL variable as dependent variable.

Group	Variable	Unadjusted model (Model-1)					Adjusted model (Model-2)				
		OR	Wald	*p*	Confidence interval 95%		OR	Wald	*p*	Confidence interval 95%	
					Lower limit	Upper limit				Lower limit	Upper limit
**Diabetes group**	**TG (mg/dL)** (Ref < 150)										
	**TG (mg/dL)** **(=** ≥ 150)	5.983	6.128	0.013	1.451	24.666	10.226	7.011	0.008*	1.829	57.161
	**HDL-C (mg/dL)** (Ref = *M* < 40, *F* < 50)										
	**HDL-C (mg/dL)** (M ≥ 40, F ≥ 50)	0.705	0.206	0.650	0.156	3.191	5.823	1.856	0.173	0.462	73.425
	**FM%**	0.941	2.158	0.142	0.868	1.021	1.102	0.543	0.461	0.851	1.428
	Constant	2.371	0.362	0.547	–	–	0.004	1.184	0.277	–	–
**Control group**	**SBP** (Ref <130)										
	**SBP** (= ≥130)	0.212	4.952	0.026	0.054	0.831	0.168	5.158	0.023	0.036	0.783
	**HDL-C (mg/dL)** (Ref = *M* < 40, *F* < 50)										
	**HDL-C (mg/dL)** (M ≥ 40, F ≥ 50)	4.164	3.418	0.064	0.918	18.891	0.658	0.164	0.686	0.086	5.010
	Constant	1.669	2.015	0.156	–	–	0.735	0.007	0.933	–	–

**Model-1:** Regression model unadjusted for independent factors.

**Model-2:** Logistic regression model adjusted for sex, age, marital status and BMI variables.

Logistic regression model performance metrics for Model-1 in the diabetes group: Nagelkerke $R^{2}$ =0.337, Cox & Snell $R^{2}$ =0.249, CCR = 0.714.

Logistic regression model performance metrics for Model-2 in the diabetes group: Nagelkerke $R^{2}$ :0.503, Cox & Snell $R^{2}$ :0.371, CCR = 0.776.

Logistic regression model performance metrics for Model-1 of the control group: Nagelkerke $R^{2}$ =0.197, Cox & Snell $R^{2}$ =0.146, CCR = 0.643.

Logistic regression model performance metrics for Model-2 of the control group: Nagelkerke $R^{2}$ =0.316, Cox & Snell $R^{2}$ 0.233, CCR = 0.732.

CCR, Correct classification rate; FM, fatt mass; HDL-C, high density lipoprotein cholesterol; OR, Odds ratio; p, Significance; SBP, systolic blood pressure.

Table 5 shows the results of the regression models in which the NEAP variable was taken as the dependent variable. When the findings of the diabetes group are examined, it is seen that the FM (%) variable does not have a significant effect on the NEAP variable in the uncorrected and corrected models (*p* > 0.05). In the findings of the control group, it was determined that the HDL-C variable did not have a significant effect on the NEAP variable in both models (p > 0.05).

**Table 5:** Regression model where the NEAP variable is taken as the dependent variable.

Group	Variable	Unadjusted Model (Model-1)					Adjusted Model (Model-2)				
		OR	Wald	*p*	Confidence interval 95%		OR	Wald	*p*	Confidence interval 95%	
					Lower limit	Upper limit				Lower limit	Upper limit
**Diabetes group**	**FM %**	0.939	3.783	0.052	0.881	1	1.068	0.428	0.513	0.877	1.302
	Constant	7.470	2.771	0.096	–	–	35.411	1.182	0.277	–	–
**Control group**	**HDL-C (mg/dL)** (Ref = *M* < 40, *F* < 50)										
	**HDL-C (mg/dL)** (*M* ≥ 40, *F* ≥ 50)	4.164	3.418	0.064	0.918	18.891	0.592	0.323	0.570	0.097	3.609
	Constant	1.669	2.015	0.156	–	–	70.766	1.555	0.212	–	–

**Model-1:** Regression model unadjusted for independent factors.

**Model-2:** Logistic regression model adjusted for sex, age, marital status and BMI variables.

Logistic regression model performance metrics for Model-1 in the diabetes group: Nagelkerke $R^{2}$ =0.103, Cox & Snell $R^{2}$ =0.077, CCR = 0.667.

Logistic regression model performance metrics for Model-2 in the diabetes group: Nagelkerke $R^{2}$ =0.217, Cox & Snell $R^{2}$ =0.162, CCR = 0.576.

Logistic regression model performance metrics for Model-1 of the control group: Nagelkerke $R^{2}$ =0.099, Cox & Snell $R^{2}$ =0.074, CCR = 0.579.

Logistic regression model performance metrics for Model-2 of the control group: Nagelkerke $R^{2}$ =0.222, Cox & Snell $R^{2}$ =0.165, CCR = 0.667.

CCR, correct classification rate, FM, fatt mass; HDL-C, high density lipoprotein cholesterol; OR, odds ratio; *p*, Significance.

## Discussion

This case–control study evaluated the potential associations between dietary acid load and CVD risk factors in individuals with diabetes. PRAL and NEAP methods were used to determine dietary acid load and the results were divided into two groups as low and high according to median values. As a result of the analysis, significant associations were found between PRAL value and TG, sex and HDL-C in the diabetes group, while only sex was associated with NEAP value. After adjusting for potential confounding factors, TG in the diabetes group and SBP in the control group were found to have an effect on the PRAL value, whereas dietary acid load, as defined by NEAP, did not. No significant association was found between other CVD risk factors and NEAP and PRAL values. These findings suggest that individuals at high risk for cardiovascular risk factors may tend to eat diets with high dietary acid load.

Common conditions such as hypertension, dyslipidemia, obesity and insulin resistance accompanying diabetes form the basis of CVD (4). Previous studies have reported associations between dietary acid load and CVD risk factors (9, 10, 18). In a study conducted in Japan in 2008 (9), while a positive association was found between PRAL values and HDL-C of the individuals, no significant relationship was found between cardiovascular risk factors such as TG, BMI and smoking. In a retrospective cross-sectional study conducted in Korea, while a significant relationship was found between PRAL and LDL-C, smoking and BMI; no significant relationship was found with WC, total and HDL-C and diabetes (18). A meta-analysis examined the association between PRAL and NEAP with CVD and lipids; six studies found a positive association between dietary acid load and lipids, while no significant association was found in other studies (10). In contrast to these studies, there are also studies reporting that there is no independent relationship between CVD risk factors and dietary acid load (19, 20). In our study, an independent relationship was found between PRAL and HDL-C, TG and FM% in the diabetes group, whereas PRAL was associated with HDL-C in the control group (Table 2). Studies have shown that protein intake and protein types can affect HDL-C levels. The amount and type of protein may have affected both PRAL and HDL-C levels (21, 22). The relationship between CVD risk factors and dietary acid load appears to be contradictory. In this study, in the diabetes group, only TG and PRAL were associated with PRAL among CVD risk factors (Table 4) but no association was observed between NEAP and risk factors (Table 5). This emphasizes the need for further longitudinal studies between dietary acid load and CVD risk factors.

In this study, a negative significant relationship was found between SBP and PRAL in the control group, and this significant relationship continued after all adjustments were made. Although the Polish study (20) and the E3N-EPIC cohort study (6) found no significant association between dietary acid load and hypertension prevalence, the Rotterdam study (23) reported that higher PRAL values were associated with blood pressure. This may be explained by the relatively low dietary acid-forming potential of individuals with diabetes in this study and other populations. Furthermore, since the use of medications that affect blood pressure and blood lipid levels of individuals with diabetes and the control group was not included as an exclusion criterion, this may constitute a potential confounding factor.

There are studies suggesting that the strength of the association between dietary acid load and both diabetes and CVD is inconsistent due to the different indices used and that gender may be a potential confounding factor in this difference. In a meta-analysis including seven prospective cohort studies, it was determined that a diet high in dietary acid load may increase the risk of diabetes, but this relationship was significant only in women. While a linear relationship was found between NEAP score and diabetes risk in women, this relationship was observed to be U-shaped in PRAL score (13). Three cohort studies conducted in diabetes showed that the association between dietary acid load score and diabetes was significant only among women (11). In a study conducted in Japan, it was observed that PRAL was associated with the risk of diabetes only in young men, but this relationship was not found between the NEAP value and diabetes. Some studies have shown that men have a higher dietary acid load and this study is similar to these findings (24). However, there are studies showing that dietary acid load values are similar in both genders (25). CVD has long been considered a condition that primarily affects men, but the actual lifetime risk of CVD appears to be similar for men and women. Moreover, a meta-analysis study found that women with diabetes have a 50% higher risk of fatal CVD compared to men with diabetes (26). Women tend to adopt a diet with a lower dietary acid load. However, considering that a diet with high dietary acid load may have an impact on the development of both CVD and diabetes, and since the prevalence of obesity is higher in women than men worldwide, gender-specific studies are required.

Due to the limited number of studies examining dietary acid load, the relationship between dietary acid load and metabolic diseases is not fully understood. It is reported that the main mechanism between dietary acid load and metabolic disease risk is insulin resistance6. However, high acidity in the blood levels may predispose to various metabolic complications such as mineral excretion, increase in blood pressure and higher cortisol secretion (27). Metabolic acidosis causes increased production of acid-forming metabolites in the body, which may lead to the release of plasma glucocorticoids, resulting in increased cortisol that supports visceral obesity and insulin resistance (28). Therefore, even in healthy individuals, there is a risk of very low degree metabolic acidosis causing hyperglycemia by causing insulin resistance (7). With the increase in dietary acid load, urinary citrate excretion decreases and it is thought that low urinary citrate excretion may be associated with insulin resistance (7, 29). Potassium and magnesium, obtained mostly from plant foods, have an important role in acid–base balance. Insufficient intake of fruits and vegetables, and therefore potassium and magnesium, directs the pH balance toward acidosis, which may disrupt the β-cell response and lead to insulin resistance (30, 31). Finally, it has been reported that minerals such as calcium and magnesium, which are necessary for the insulin response, may cause significant insulin dysfunction due to increased urinary excretion (32).

This study has some strengths and limitations. If we look at the strengths of the study, first of all, this study is the first study in our country to examine the relationship between CVD risk factors and dietary acid load obtained from the diet in individuals with type 2 diabetes. Secondly, nephropathy can develop in individuals with diabetes, so the participants’ kidney functions, which are critical in determining acid–base homeostasis, were controlled, therefore we attempted to reduce the impact of chronic metabolic acidosis or alkalosis by excluding individuals with chronic kidney disease, liver failure or cirrhosis, congestive heart failure or a history of CVD, and chronic obstructive pulmonary disease. Despite the strong aspects, the study also has some limitations: first, given the case–control design of the study, we could not conclude a causal relationship as to whether a high dietary acid load leads to the development of cardiometabolic diseases or vice versa. Therefore, interventional studies are needed to determine whether dietary acidity has an effect on the development of cardiometabolic diseases. Secondly, individuals’ dietary intakes were recorded with a 3-day food consumption record. Inaccurate reporting of dietary intake, especially by obese individuals, is an important problem with diet assessment methods based on self-reports. Also, compared to direct observation of food intake, self-reporting typically results in incomplete reporting of food intake. Third, PRAL and NEAP values were estimated from self-reported 3-day dietary intake and were not evaluated objectively. However, changes in dietary patterns over time, the actual nutritional composition of specific meals, preparation methods, and absorption of nutrients in the gastrointestinal tract are not taken into account by equations that measure dietary acid load, such as PRAL and NEAP. Dietary PRAL and NEAP scores are frequently used in epidemiological studies and although they are highly correlated with measured acid load, they may be affected by inaccurate nutritional reports (14, 15).

## Conclusion

In conclusion, after correcting for possible confounding factors, we found that higher PRAL value was associated with higher TG in individuals with diabetes, but we did not observe any association between NEAP value and risk factors. Aiming for an improvement in dietary acid–base balance may be a useful strategy for preventing cardiometabolic disorders. However, further prospective studies are needed to observe the effects of dietary acid–base load on diabetes and cardiometabolic risk factors better.

## Data Availability

The datasets presented in this article are not readily available because of privacy or ethical restrictions. Requests to access the datasets should be directed to SG, sedef.gungor@atilim.edu.tr.
